# Epigenetics and bronchopulmonary dysplasia: unraveling the complex interplay and potential therapeutic implications

**DOI:** 10.1038/s41390-024-03268-3

**Published:** 2024-05-16

**Authors:** Rui Song, Vineet Bhandari

**Affiliations:** 1 Lawrence D. Longo, MD Center for Perinatal Biology, Department of Basic Sciences, Loma Linda University School of Medicine, Loma Linda, CA USA; 2 Division of Neonatology, The Children’s Regional Hospital at Cooper/Cooper Medical School of Rowan University, Camden, NJ USA

Despite advances in neonatal medicine, bronchopulmonary dysplasia (BPD) remains a major challenge for preterm infants.^1^ BPD is characterized by impaired alveolar and vascular development, which can lead to long-term respiratory (and neurodevelopmental) morbidity and increased healthcare costs.^1,2^ BPD occurs secondary to genetic-environmental interactions,^3^ with lung immaturity (due to preterm birth) being a critical element in its’ pathogenesis.^2^ Although the etiology of preterm birth is multifactorial and not fully understood, recent research has implicated epigenetic alterations in the dysregulation of key pathways involved in fetal growth, immune function, and stress response,^4–6^ contributing to the increased vulnerability of preterm infants to adverse outcomes (such as BPD) with a lifetime of consequences.

There is a growing body of evidence that DNA methylation is one of the most important epigenetic mechanisms involved in preterm birth. In DNA methylation, a methyl group is added to the cytosine residue of CpG dinucleotides, resulting in gene silencing.^7^ It has been reported that preterm infants have differentially methylated regions (DMRs) in their placentas and cord blood compared to full-term controls.^8,9^ These DMRs have been associated with genes involved in various biological processes, such as immune regulation, cell adhesion, and signal transduction. Preterm infants display higher levels of DNA methylation in inflammatory-related genes compared to term infants.^10^ Inflammation remains a keystone in the pathogenesis of BPD, responsible for the initiation and progression of the lung injury response and the subsequent modification of the pulmonary pathology by the imbalance in the lung growth and healing process, favoring lung repair.^11^ The findings suggest that epigenetic dysregulation of inflammatory-related genes may contribute to the heightened and persistent inflammatory response observed in preterm infants and their increased risk for developing inflammatory complications such as BPD.^11^

Aside from DNA methylation, histone modifications and non-coding RNAs have also been implicated in preterm birth pathogenesis. By altering chromatin structure and accessibility, histone modifications, such as acetylation and methylation, can affect gene expression.^12^ The modification patterns of histones in preterm infants differ from those in full-term infants, particularly in genes linked to inflammation and oxidative stress.^13,14^ There is also evidence that altered patterns of non-coding RNAs such as microRNAs (miRs), and long non-coding (lnc)RNAs, may regulate gene expression and cellular function in preterm birth and BPD.^15,16^ An analysis^17^ identified a number of miRs that differ in expression between preterm and full-term infants, including miR-210, which has been associated with hypoxia and oxidative stress.^18,19^ Moreover, epigenetic modifications can be reverted, making them an attractive therapeutic target. Animal models of preterm birth have shown that histone deacetylase inhibitors reduce inflammation and promote lung maturation.^20,21^ In addition, miR mimics and inhibitors are potential strategies for modulating gene expression and improving the pulmonary pathology in BPD.^22,23^

In their paper, Wang et al.^24^ do a comprehensive review of DNA methylation, histone modifications, miRs, lncRNAs, and circular (circ)RNAs role in BPD. They discuss the different sources (lung tissue from mice in experimental BPD models, and cord blood as well as buccal samples from human preterm infants) that have been used to detect the epigenetic changes. In addition, they have highlighted mesenchymal stem cell (MSC)-derived exosomes as one of the most promising therapeutic approaches for BPD. A variety of bioactive molecules are present in these extracellular vesicles, including non-coding RNAs that are capable of modulating recipient cells’ epigenetic landscapes.^25^ Exosomes derived from MSCs have been shown to reduce hyperoxia-induced lung injury and improve alveolarization in animal models of BPD.^26–28^ The therapeutic potential of exosomes lies in their ability to deliver epigenetic modulators and alter gene expression profiles of target cells, thereby promoting lung regeneration and repair.

Most of the data generated, as reported in the review by Wang et al.^24^, have been generated using animal models. So, while such preclinical findings are promising, translating them into clinical practice remains challenging. Some suggested approaches that could be applied to obtain a deeper understanding of the epigenetic landscape of the developing lung, would include advanced imaging techniques such as single-cell sequencing and spatial transcriptomics. Using these techniques, we may be able to identify specific cell types and regions that are most affected by aberrant epigenetic modifications, allowing targeted therapies to be developed. This is important as the lung is made up of multiple cell types. From the scientific perspective, while identification of molecular signaling pathways in a specific cell type is helpful, it is the totality of the effect on lung development and response to injury that determines the final pulmonary phenotype of BPD. As a step towards understanding the interaction and integration of molecular signaling in the whole organ, it is possible to simulate lung development by using organoids or 3D cell culture models that mimic complex cellular interactions and environmental factors. It may prove beneficial to use these models to study epigenetic mechanisms underpinning BPD and to test possible therapeutic interventions. For translational relevance, it would be critically important to determine if the epigenetic modification is also detected in developmentally appropriate human pulmonary tissues (tracheal aspirates, lung tissue) before considering clinical trials. Access to such samples is crucial to help decide which potential therapies can be moved from the bench closer towards the bassinet in the neonatal intensive care unit. In addition, if epigenetic therapies directed towards prevention of BPD are to be considered in the antenatal period, these must be carefully evaluated for safety and efficacy during pregnancy and fetal development to minimize potential risks.^29^

The future of epigenetics research needs to include multi-omics data, longitudinal studies, and standardized approaches for epigenetic profiling and data analysis. Further, developing personalized medicine approaches based on BPD pulmonary phenotypes ^30^ that are impacted by specific epigenetic modifications needs to be developed in experimental in vitro and in vivo BPD models and also detected in human lung BPD tissues prior to testing for their therapeutic potential in the clinical arena. The interaction of genetic, epigenetic, and environmental factors in the context of preterm birth must be further studied to develop safe and effective prevention and treatment strategies. Epigenetic research can help by identification of potential therapeutic targets that can, after appropriate clinical testing, be integrated into clinical practice, thus reducing the global burden of BPD, culminating in improved health outcomes of preterm infants.
